# Simple Sequence Repeat Gene Polymorphisms in Yellow‐Rumped Flycatcher With Gender‐Specific Associations and Personality Variations

**DOI:** 10.1002/ece3.72991

**Published:** 2026-02-03

**Authors:** Mingju E, Xudong Li, Yu Zhang, Junlong Yin, Kevin H. Mayo, Haitao Wang

**Affiliations:** ^1^ School of Life Sciences Changchun Normal University Changchun China; ^2^ School of Ecology and Environment Baotou Teachers' College Baotou China; ^3^ Zhongshan Sanxin School International Department Zhongshan China; ^4^ Department of Biochemistry, Molecular Biology & Biophysics University of Minnesota Health Sciences Center Minneapolis Minnesota USA; ^5^ Jilin Engineering Laboratory for Avian Ecology and Conservation Genetics, School of Life Sciences Northeast Normal University Changchun China

**Keywords:** gender‐specific, gene polymorphisms, personality, simple sequence repeat, SSR, yellow‐rumped flycatcher

## Abstract

This study explores the genetic and physiological facets of personality variations in the yellow‐rumped flycatche*r* ( *Ficedula zanthopygia* ), with a focus on potential sex‐specific associations between simple sequence repeat (SSR) polymorphisms, body condition index (BCI) and behavioral traits. During the 2020 breeding seasons at Zuojia Nature Reserve, northeast China, we conducted field investigations using several stress tests to quantify personality as reflected in breathing rates. This metric demonstrated significant reproducibility between life stages, thereby validating its use as a reliable association with individual boldness. We further examined the influence of genetic diversity by genotyping 10 highly polymorphic SSR loci and calculating individual heterozygosity. As a reflection of stronger personalities, we found significant associations between individual heterozygosity and breathing rates in female adults, with greater heterozygosity correlated with lower breathing rates. The opposite pattern was observed in male nestlings, and no significant correlations were observed in male adults or male chicks. In addition, the BCI tended to be negatively correlated with breathing rates in female adults, suggesting that individuals with better body conditions were less fearful. These findings underscore the importance of genetic diversity and body condition in modulating personality traits, particularly in females. Overall, our results highlight the likelihood that the sex of these birds underlies their behavioral variations. Moreover, this study provides insight into the genetic basis of personality in cavity‐nesting birds and emphasizes the need for further research to elucidate specific genetic pathways that influence these traits.

## Graphical abstract

Our research provides novel insights into the genetic and physiological underpinnings of personality variation in wild birds, specifically focusing on the yellow‐rumped flycatcher ( *Ficedula zanthopygia* ). Through rigorous field investigations and genetic analyses, we demonstrate that female flycatchers with higher genetic diversity and better body condition exhibit stronger personalities, as measured by lower breathing rates. This finding highlights the importance of genetic diversity and body condition in modulating behavior and suggests sex‐specific mechanisms underlying personality traits in birds. Our study not only fills a gap in the literature regarding the genetic basis of personality in wild cavity‐nesting birds but also underscores the need for further research into the specific genetic pathways influencing these traits.

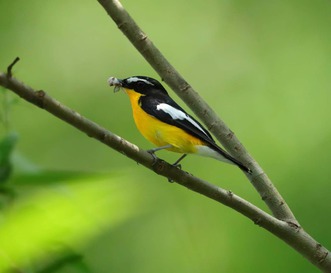

## 1 Introduction

Variation in individual animal behavior arises from genetic factors, environmental influences, and learning experiences (Breed and Sanchez 2010; Stamps and Groothuis 2010). Behavioral differences among individuals within the same population—termed animal personality—are observed through time and context (Sih et al. 2004; Royle et al. 2010), including traits such as exploration, aggression, and neophobia (fear of new objects or situations) (Bibi et al. 2022). Behavioral traits often differ among individuals within the same population (Sloan Wilson et al. 1994). This, in turn, has a genetic basis (Laine and van Oers 2017). Although associations between genes and behavior depend on the environment (Virolainen et al. 2023), recent studies have suggested that personality (behavioral variation) is not random and is influenced by genetic factors, including heterozygosity (Fidler et al. 2007; Grunst et al. 2021). For example, the serotonin transporter gene (SERT) has been reported to likely affect neophobia and exploratory behavior (Grunst et al. 2021), and the dopamine receptor D4 gene (Drd4) is associated with fearless behavior (Timm et al. 2015). However, these behaviors and genes vary within specific populations, possibly resulting from environmental‐genotypic interactions or differences in genetic polymorphisms (Li et al. 2024).

Previous studies have demonstrated that personality affects a variety of life‐history decisions in birds, such as survival (Richardson et al. 2019), reproduction (Li et al. 2024), nest defense (Hollander et al. 2008), and dispersal (Luna et al. 2019). In birds, personality is related to hormone levels (Ruuskanen et al. 2018; Mengelkoch et al. 2021) and genetics (Fidler et al. 2007). Moreover, in seeking to understand evolutionary changes in phenotypes, one needs to investigate associations among genes, behaviors, and fitness hormone levels within the same population (Bergmüller et al. 2010; Van Oers et al. 2005). Body condition may also influence the development of individual personalities (Moiron et al. 2019; Vágási et al. 2021), and that personality can influence reproductive fitness in flycatchers (Tamin et al. 2023). However, correlations among these traits have yet to be thoroughly investigated at the genetic level. In addition, difficulties in tracking, tagging, and manipulating wild birds in order to measure their behavior have made collecting data relatively difficult. Therefore, there remains a lack of empirical data concerning the genetic impact on personality in wild cavity‐nesting birds (Grunst et al. 2021; Timm et al. 2015).

The yellow‐rumped flycatcher ( *Ficedula zanthopygia* ) is a migratory cavity‐nesting bird that exhibits solitary nesting, biparental care, and extra‐pair paternity (E et al. 2021). Its ready use of artificial nest boxes makes it a highly tractable model for field‐based behavioral and genetic studies. Its nest‐site fidelity and accessibility allow for repeated physiological and behavioral measurements—such as standardized stress response assays—while its breeding system promotes genetic diversity within populations, which is why we used this species as a relevant model for investigating whether simple sequence repeat (SSR) polymorphisms and body condition indexes (BCI) are correlated with personality variations in this closed‐nesting avian population. For this study, we used breathing rate as a measure of personality because it can be accurately measured with good reproducibility (Lou et al. 2023). Although breathing rate is thought to be a physiological characteristic that reflects stress response (Guindre‐Parker et al. 2022; Pearmain‐Fenton et al. 2022), many studies have related a low breathing rate with stronger boldness (Haigh et al. 2017; Peleg et al. 2014). In fact, Zhao et al. (2016), has reported that birds with a lower breathing rate are indeed bolder than those with a higher breathing rate. Given that there may also be sex differences in parental behavior and personality (Krams et al. 2014; Lou et al. 2021; Wei et al. 2014), we further hypothesized that the relationship between personality and sex differs between males and females. Overall, our study has increased our understanding of genetic links to personality in cavity‐nesting wild birds.

## 2 Methods and Materials

### 2.1 Subjects and the Study Site

This study was conducted on the migratory yellow‐rumped flycatcher during the 2020 breeding season at Zuojia Nature Reserve (126°1′–127°2′ N, 44°6′–45°5′ E) in Jilin Province, northeast China. Each year, these birds populate ~450 nesting boxes. For this study, we used wood‐board artificial nesting boxes (12.5 × 12.5 × 25 cm) with a circular hole of 4 cm at the front of the box. Nesting boxes were placed at 3 to 4 m above ground on trees populated with various bird species. Distances between boxes were held at 30 to 50 m. Nests were visited every 1–2 days to monitor the settling order between nearest neighbors and to determine egg‐laying and hatching dates. We considered a box to be occupied when nesting materials were continuously present. Female yellow‐rumped flycatchers usually lay one egg per day (clutch sizes range from 5 to 8 eggs) per season, and they usually complete nest building within 3 to 4 days prior to laying eggs. The geographical position of each nest was recorded using a GPS unit (N400PLUS, BHCnav, Beijing, China).

### 2.2 Measurement of Personality Traits

When nestlings were 6 days old, we first fitted each chick with a unique color ring, after which adult birds were captured by using a spring trap between the hours of 08:00 and 16:00, with the adult bird removed and placed in a cotton bag. The breathing rate was measured by using the handling stress (HS) test, and we recorded the total number of breast movements within 1 min divided into four bouts of 15 s each in order to track possible patterns on a finer scale (Fucikova et al. 2009). The regression coefficient, *k*, was used to represent the degree of change in breathing (i.e., breathing slope). Following studies on adults, we performed the HS test on the chicks by taking nestlings from their boxes, placing them in cotton bags, and blocking the entrance to the nest box with gloves to stop parents from entering the box. During the HS test, we held the chick with one hand and gently covered its head with the other. The chick remained in this position until the breathing rate could be calculated by observing chest movement. For each individual, the measurement was performed four consecutive times for 15 s each with individual chicks being isolated in dark cloth bags for at least 10 min. Since we often removed all chicks from a brood at once, only one (randomly selected) chick at a time was tested for HS, and the rest were left on small pads made of cotton until all tested chicks had been placed in dark‐colored textile bags. When all chicks from a brood had been examined, their weights and tarsus lengths were measured. After all chicks were returned to the nest box, the glove blocking the entrance was removed to allow the parents to resume normal brooding activities.

To calculate the reproducibility of the HS test, we repeated the test when offsprings were 11 to 12 days old. Due to the difficulty in capturing adult birds during the brooding period and the risk of their abandoning the nest caused by repeated capture, we did not conduct repeated measurements of the respiratory rate of the adult birds. The body mass (to the nearest 0.1 g) and tarsus length (to the nearest 0.1 mm) were measured prior to release.

### 2.3 SSR Genotyping

The DNA from adult and chick birds was extracted from their blood using the UNIQ 10‐column animal genomic DNA isolation kit (SK1206, Sangon, Shanghai). PCR amplification was performed on 10 highly polymorphic loci Fhy341, Fhy463, Fhy429, Fhy321, Fhy415, Fhy458, Fhy453, Fhy444, Fhy344, and Fhy450. PCR amplification of the 10 highly polymorphic loci was used to analyze the heterozygosity of microsatellite genes (E et al. 2017). The sex of the chicks was determined molecularly using the primer set sex1 (5′‐CTCC CAAG GATG AGAA ACTG TGCA AAAC AGGT A‐3′) and sex‐mix (5′‐CCTT CRCT KCCA TTRA AGCT RATC TGGA AT‐3′) (Wang et al. 2010).

### 2.4 Statistical Analyses

To calculate breathing rates of the yellow‐rumped flycatchers over the 1 min examination, we divided that 1 min into four values of 15 s each. The breathing frequency for 15 s and the number of experiments (1, 2, 3, 4) were input into the regression Equation (1) to find the slope *k*, i.e., the regression coefficient.

(1) $$k=\frac{\sum x_{i}y_{i}-4\bar{x}\bar{y}}{\sum x_{i}^{2}-4{\bar{x}}^{2}}$$

Here $x_{i}$ represents the breathing frequency within 15 s; $y_{i}$ represents the number of groups in the experiment; $\bar{x}$ represents the average number of breathing frequencies over the 15 s, and $\bar{y}$ represents the average number of experimental groups 1–4 ($\bar{y\;}=2.5$). The value of *k >* 0 indicates that a bird's breathing rate has increased over the experiment time period, and when *k* < 0, the bird's breathing rate has a downward trend.

Because not all individual birds were typed using the same set of microsatellite markers, we calculated the standardized individual heterozygosity (i.e., proportion of heterozygous sites/average heterozygosity of typed sites). This approach was used for all analyses and is termed “individual heterozygosity” for simplicity. We calculated the mean $d^{2}$ value to represent individual genetic diversity (i.e., the square of the length difference between alleles at a locus) (Foerster et al. 2003).

We used the Kolmogorov–Smirnov test to evaluate the normality of the breathing rate of the chicks, and the data did not conform to normality (*D* = 0.3208, *p* < 0.01). Therefore, we used the Wilcoxon signed ranks test to analyze whether there were differences in respiratory rates of the chicks at 6 and 12 days to determine reproducibility of a personality (Applegate et al. 2003).

The Dunn test was employed to examine whether differences existed in breathing rate among individuals of different sexes and ages. Where significant differences were detected, post hoc comparisons were conducted, with *p*‐values adjusted using the Bonferroni correction method. We also used generalized linear models (GLMs, family = Gamma(link = “log”)) to analyze effects of individual heterozygosity and body condition index (BCI) on the personality (breathing rate) of female and male adult birds and generalized linear mixed models (GLMMs, family = Gamma(link = “log”)) for chicks. For all models, breathing rate data were shifted upward by three units to meet the distributional assumptions of the Gamma distribution. Breathing rate data were shifted upward by three units to conform to the Gamma distribution. BCI was represented by the ratio of body weight to tarsus length (Stauss et al. 2005). In models for adult birds (analyzed separately by sex), individual heterozygosity and BCI are fixed factors and breathing rates of female and male adults were taken as dependent variables. For chicks (analyzed separately by sex), we used GLMMs that additionally incorporated Nest Box ID as a random intercept to account for shared environmental effects among siblings. Fixed effects and the dependent variable were specified as in adult models. The Akaike information criteria (AIC) was employed to evaluate the model fit. AIC comprehensively considers the goodness‐of‐fit and parameter complexity of the model; generally, a lower AIC value indicates a better comprehensive fitting effect of the model.

We performed all statistical analyzes using the software package R v4.2.3 with the R‐package “lme4” and SPSS 22.0 (IBM SPSS Statistics 2013). All statistical tests were two‐tailed. We considered values to be statistically significant when α < 0.05.

## 3 Results

We examined a total of 88 adult flycatchers using HS testing, with 46 females and 42 males. A total of 73 chicks were subjected to HS testing, of which 40 were females and 33 were males. There was no significant difference in respiratory rates between chicks at 6 and 12 days, indicating that the character of flycatchers was stable (Wilcoxon signed ranks test: *V* = 1301, *p* = 0.73).

Ten pairs of microsatellite loci were detected in 105 adults and 80 nestlings. The number of microsatellite alleles ranged from 6 to 24; the average number of alleles at each locus was 13.62 ± 4.513, and the polymorphism content was 0.832 ± 0.021. The non‐pro exclusion rate was 99.99%, and the cumulative non‐parental exclusion rate for multiple sites was 0.001.

The average value of individual heterozygosity of female adults was 0.003 ± 0.003 (SD), and the average value of individual heterozygosity of male adults was 0.003 ± 0.0005 (SD). For chicks, the average value of individual heterozygosity of females was 0.002 ± 0.0005 (SD), and the average value of individual heterozygosity of males was 0.003 ± 0.0004 (SD).

**FIGURE 1:**
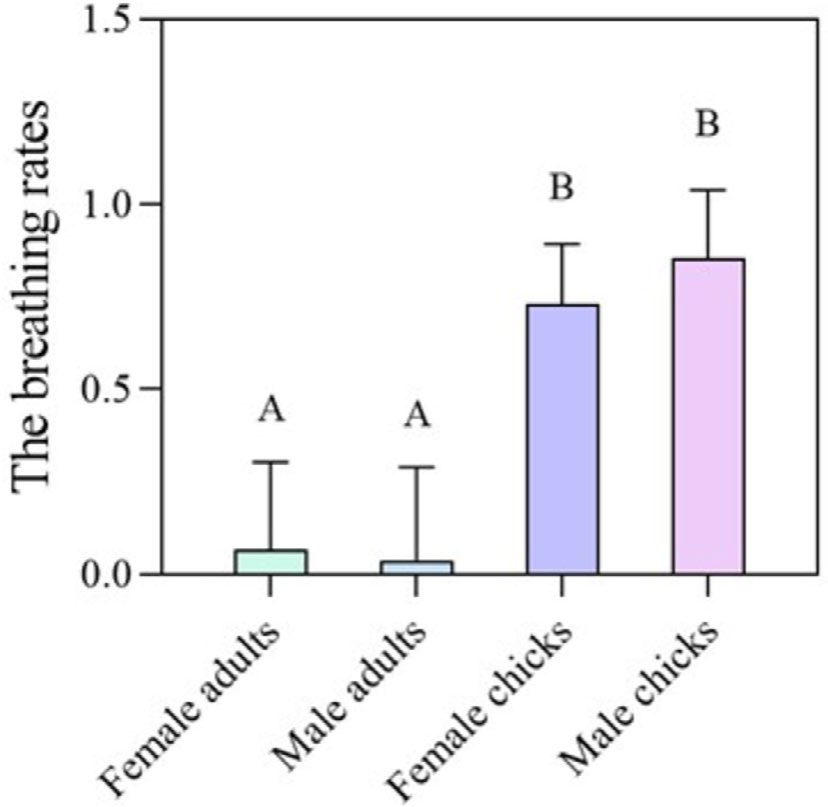
The effects of different ages and sexes on the breathing rate of yellow‐rumped flycatchers. Different letters above the bars indicate significant differences in breathing rates among individuals of different ages and sexes (post hoc test: *p*_adj_ ≤ 0.05).

The breathing rates of female adults were significantly lower than those of female chicks (dunn.test, *p* = 0.01) and male chicks (*p* = 0.01); the breathing rates of male adults were also significantly lower than those of female chicks (*p* = 0.06) and male chicks (*p* = 0.03, Figure 1). However, there was no significant difference in breathing rates between adult females and adult males (*p* = 1), nor between female chicks and male chicks (*p* = 1).

**FIGURE 2:**
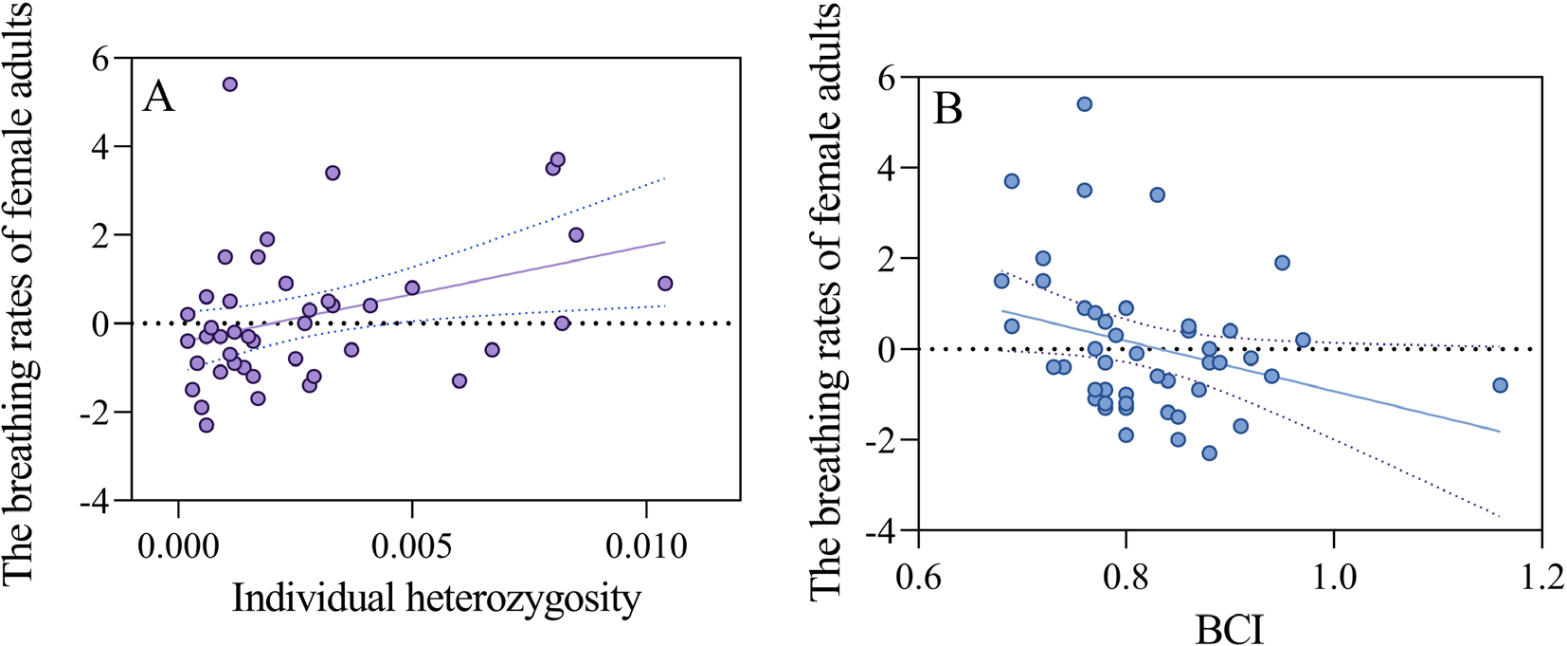
Relationship between individual heterozygosity and breathing rate (A), as well as BCI and breathing rate (B) in female adults.

The breathing rates of female adults were significantly influenced by individual heterozygosity (GLMM, *χ* ^2^ = 3.88, df = 1, *t* = 1.95, *p* = 0.049), whereas BCI showed a non‐significant trend (*χ* ^2^ = 2.96, df = 1, *t* = −1.65, *p* = 0.09, Figure 2, Table S1). On the other hand, breathing rates of male adults were not influenced by individual heterozygosity (GLMM, *χ* ^2^ = 0.28, df = 1, *t* = −0.52, *p* = 0.60) and BCI (*χ* ^2^ = 1.10, df = 1, *t* = −0.36, *p* = 0.75).

**FIGURE 3:**
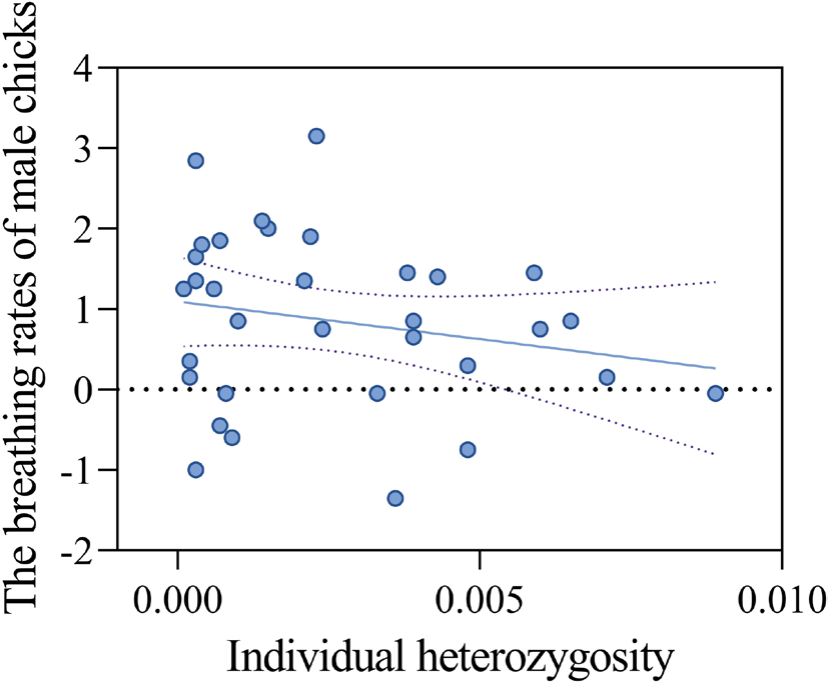
Relationship between individual heterozygosity and breathing rate in male chicks.

Even though breathing rates of male chicks were significantly influenced by individual heterozygosity (GLMM, *χ* ^2^ = 4.46, df = 1, *t* = −2.11, *p* = 0.03, Figure 3, Table S1), BCI values were not (*χ* ^2^ = 0.19, df = 1, *t* = 0.43, *p* = 0.67). However, breathing rates of female chicks were not influenced by individual heterozygosity (GLMM, *χ* ^2^ = 1.36, df = 1, *t* = 1.17, *p* = 0.24) or BCI values (*χ* ^2^ = 2.13, df = 1, *t* = 1.46, *p* = 0.14).

## 4 Discussion

The purpose of this study was to explore effects of individual heterozygosity and BCI on a simple personality test (breathing rate) of individual birds in the wild. Here we discovered that with female adults and male chicks, there was a correlation between individual heterozygosity and breathing rate. However, BCI values of female chicks did not significantly affect their breathing rate. Our study showed that HS was reproducible across life stages and that individual heterozygosity and BCI were related to personality, at least to some extent. To our knowledge, this relationship had not been reported previously in this bird species.

In natural populations, there are few known examples of polymorphisms in several neurotransmitter‐related genes associated with variations in avian personality traits. In great tits ( *Parus major* ), polymorphisms have been identified in neurotransmitter‐associated genes (e.g., Drd4 SNP830), with the allele frequency of individuals with slow early exploratory behavior being significantly lower than that in individuals with fast early exploratory behavior (Fidler et al. 2007). Under mild stress, the behavior showed a consistent pattern, with similar changes in exploratory behavior, boldness, and aggression (Drent et al. 2003). Thus, higher genetic heterozygosity in females, but not in males, may be bolder in flycatchers.

Compared with adult birds, chicks exhibit an incompletely developed respiratory system, characterized by smaller lung volumes and lower gas exchange efficiency (Maina 2015). Furthermore, as chicks are in a phase of rapid growth and development, they undergo vigorous vital activities such as cell division and tissue organogenesis, leading to a basal metabolic rate that is substantially higher than that of adult birds (Nagy 1987). Consequently, chicks display a higher breathing rate than adult birds. In female adults, greater individual heterozygosity was correlated with a faster breathing rate. Breathing rate is an indicator of the acute stress response of birds and can reflect an individual bird's personality (such as exploratory tendencies and neophobia) (Fucikova et al. 2009). It is generally believed that individual birds with slower breathing rates are more bold (Carere and van Oers 2004). Previous studies have shown that birds with low heterozygosity exhibit shorter flight initiation distances (FIDs), indicating that these individuals possess heightened sensitivity to potential threats and correspondingly display more proactive anti‐predator behavior (Jiang and Møller 2017). Thus, female adults with low individual heterozygosity may exhibit a less bold behavioral phenotype. Notably, this association found in females contrasts with the commonly observed links between heterozygosity and sexually selected traits in males (e.g., territory size; Seddon et al. 2004), suggesting that the influence of genetic diversity on behavior may vary depending on sex and selective pressures.

On the other hand, the breathing rates of male adults were not influenced by heterozygosity. Males usually need to compete for nesting sites, territories, and mating rights, whereas individuals with higher heterozygosity have obvious advantages over individuals with low heterozygosity in this competitive situation (García‐Navas et al. 2009; Isomursu et al. 2012). Thus, individuals with low heterozygosity must cautiously assess potential risks from the environment and competitors, and therefore exhibit a similar behavioral phenotype as those with high heterozygosity.

These sex‐specific behavioral variations may be explained by differences in parental care by male and female birds. In this regard, some studies suggest that female behavior in terms of parental care is more genetically influenced than male behavior. For example, females from different lineages show more pronounced differences in social status in slow‐ and fast‐exploration‐selected lines of great tits (Carere et al. 2005).

Here, we found that BCI was negatively correlated with breathing rates in female adults. This suggests that larger individuals may have lower relative breathing rates compared to smaller individuals. Our results may explain whether the expression of bird antipredator behavior is affected by body condition, as is the case in other animal species (Lindström et al. 2003; López et al. 2005). We suggest that individual birds in poor health are shier of facing simulated predators. Interestingly, however, this relationship was not found in males. Previous studies found that females usually have a greater daily energy expenditure than males in this and other songbird species (Verhulst and Tinbergen 1997; Linek et al. 2021; Tinbergen and Dietz 1994). Therefore, BCI has a more significant impact on females compared to males.

Contrary to results on female adult birds, we found that the higher the individual heterozygosity of male chicks, the slower their breathing rate, suggesting that they are bolder. Adult individuals and chicks occupy distinct life‐history stages, with significantly divergent selective pressures. Adults must cope with a complex and variable external environment, where bold behavioral phenotypes may substantially increase their exposure to potential threats (e.g., predation risk and resource competition). In contrast, chicks are confined to the nest during their early developmental stage, with the nest structure providing a relatively safe microenvironment that significantly reduces the risk of danger (Fargallo et al. 2022). Chicks with greater heterozygosity and endowed with superior genetic vigor can effectively acquire intra‐nest environmental information via bold exploration. In this regard, they gain an advantage in competing for parental care resources (e.g., feeding opportunities and parental attention) that enhances their growth and development, as well as post‐fledging survival probability. This echoes the view that heterozygosity can indirectly affect fitness through behaviors such as foraging (Liebgold et al. 2018), and further reveals that the direction of such indirect effects can be strongly modulated by ontogenetic stage.

However, we detected no correlation between individual heterozygosity and breathing rate in female chicks. Previous research has shown that compared with male chicks, female chicks exhibit a lower growth rate and smaller body size (Segura et al. 2024). This difference suggests that female chicks may adopt a distinct energy allocation strategy, prioritizing developmental processes even at a slower growth rate over the expression of bold behavioral phenotypes. Correspondingly, their breathing rate—a proxy for personality—showed less differentiation in relation to genetic diversity, resulting in the undetected correlation between heterozygosity and breathing rate.

In summary, our study has revealed a relationship between genetic diversity, body condition, and personality in the yellow‐rumped flycatcher. The concept of genetic heterozygosity in birds emphasizes the importance of genetic diversity in shaping personality traits. The presence of multiple alleles at a genetic locus can provide a broader range of behavioral responses to environmental challenges. Collectively, our results indicate that associations between heterozygosity and behavioral traits are not fixed but exhibit complex sex‐ and age‐specific patterns. Further research is needed to uncover the mechanisms behind these patterns and their ultimate fitness consequences.

## Author Contributions

**Mingju E:** data curation (equal), formal analysis (equal), funding acquisition (equal), investigation (equal), methodology (equal), writing – original draft (equal). **Xudong Li:** formal analysis (equal), investigation (equal). **Yu Zhang:** conceptualization (equal), formal analysis (equal). **Kevin H. Mayo:** validation (equal), writing – review and editing (equal). **Junlong Yin:** data curation (equal), investigation (equal), methodology (equal), validation (equal). **Haitao Wang:** conceptualization (equal), funding acquisition (equal), supervision (equal), writing – review and editing (equal).

## Funding

This work was supported by the Scientific Research Fund of Jilin Provincial Education Department JJKH20261560 to M.E. and the High‐level Startup Talents Introduced Scientific Research Fund Project of Baotou Teacher's College, China (No. BTTCRCQD2024‐C34) to X.L.

## Ethics Statement

All birds were captured under a bird‐ring license issued by the China Bird Banding Center. This field survey was approved by Zuojia Nature Reserve and Forestry Bureau of Jilin Province (Approval No.: (2006) 178). Experimental methods were approved by the National Animal Research Administration of Northeast Normal University (approval number: NENU‐20080416). There are no records of deaths or nest destruction as a result of this study.

## Conflicts of Interest

The authors declare no conflicts of interest.

## Supporting information


**Table S1:** ece372991‐sup‐0001‐TableS1.docx.

## Data Availability

All the required data are uploaded as Supporting Information.
