# Supplementary material for: Simple Sequence Repeat Gene Polymorphisms in Yellow‐Rumped Flycatcher With Gender‐Specific Associations and Personality Variations
Source: Ecol Evol. 2026 Feb 3;16(2):e72991. doi: 10.1002/ece3.72991 (PMC12865505; doi:10.1002/ece3.72991)
Supplement: Supplementary file 1 — Table S1: ece372991‐sup‐0001‐TableS1.docx. [file ECE3-16-e72991-s001.docx]

**Table S1 Factors influencing breathing rate of Yellow-rumped Flycatchers**

|  | Estimate±SE | χ2 | *df* | *t* value | *P* value | AIC |
| --- | --- | --- | --- | --- | --- | --- |
| **Female adults** | | | | | | |
| Individual heterozygosity | 54.47±27.9 | 3.88 | 1 | 1.95 | 0.05 | 149.23 |
| BCI | -1.36±0.83 | 2.96 | 1 | -1.65 | 0.09 |  |
| **Male adults** | | | | | | |
| Individual heterozygosity | -31.12±60.13 | 0.28 | 1 | -0.52 | 0.6 | 195.22 |
| BCI | -0.71±1.97 | 0.1 | 1 | -0.36 | 0.75 |  |
| **Female chicks** | | | | | | |
| Individual heterozygosity | 21.40±18.35 | 1.36 | 1 | 1.17 | 0.24 | 109.58 |
| BCI | 0.30±0.21 | 2.13 | 1 | 1.46 | 0.14 |  |
| **Male chicks** | | | | | | |
| Individual heterozygosity | -31.45±14.88 | 4.46 | 1 | -2.11 | 0.03 | 87.06 |
| BCI | 0.15±0.34 | 0.19 | 1 | 0.43 | 0.67 |  |
